# Exploring Distinct Profiles in Paediatric Bioethics—An Analysis of Proactive and Reactive Approaches by Adults

**DOI:** 10.3390/children12020120

**Published:** 2025-01-23

**Authors:** Erika Rigotti, Marco Zaffanello, Sara Patuzzo Manzati, Giulia Adele Dinicola, Giorgio Piacentini, Giulia Rodella, Lucia Pozzuoli, Giovanni De Manzoni

**Affiliations:** 1 Department of Paediatrics, Woman’s & Child’s University Hospital, I-37126 Verona, Italy; erika.rigotti@aovr.veneto.it (E.R.); giulia.rodella@aovr.veneto.it (G.R.); lucia.pozzuoli@aovr.veneto.it (L.P.); 2 Department of Surgery, Dentistry, Gynecology and Paediatrics, University of Verona, I-37129 Verona, Italy; sara.patuzzomanzati@univr.it (S.P.M.); giorgio.piacentini@univr.it (G.P.); giovanni.demanzoni@univr.it (G.D.M.); 3 Cleveland Center for Bioethics, Cleveland Clinic, Cleveland, OH 44195, USA; dinicog@ccf.org

**Keywords:** bioethics, children, end of life, medical ethics, paediatric bioethics, palliative care, questionnaire, survey

## Abstract

Background/Objectives: The field of paediatric bioethics addresses ethical issues in paediatric care, where parental authority often guides medical decisions, but children’s preferences should also be considered. Promoting ethical awareness among minors can help them understand complex issues. This study aimed to analyse how sociodemographic, educational, and experiential factors shape adult perspectives on paediatric bioethical issues, particularly concerning pain and death. Methods: A questionnaire was developed to collect adult views of bioethical issues. The online questionnaire was disseminated via e-mail or WhatsApp. It started with an initial group of known individuals and then expanded hierarchically to include contacts of friends. Participants completed an online questionnaire, and data were analysed using SPSS version 22.0 for Windows. Clustering analysis identified two distinct groups. Results: This research focused on Italian adults (N = 889) aged 18 and over. Cluster 1, predominantly female (78.3%) and more highly educated (38.6% with postgraduate degrees), exhibited greater experience with paediatric bioethical issues (81.1%). This group favoured a collaborative approach, supporting shared training among adults (92.3%) and advocating for gradually addressing bioethical issues during childhood (84.3%). Cluster 2, with a higher proportion of males (31.5%) and parents (75.1%), showed lower educational levels (3.0% with middle school education) and less experience with bioethical concerns (93.5%). This group preferred a reactive, situation-specific approach to these issues. Conclusions: This study showed two distinct adult profiles regarding how they approach paediatric bioethics. The first group adopts a preventive and collaborative strategy, while the second group is more reactive and situation-driven. These findings can guide the development of tailored educational programmes to improve discussions about sensitive topics such as pain, incurability, and death in paediatric care.

## 1. Introduction

Bioethics emerged as a discipline in the 1970s, and it investigates moral inquiry to identify ethical principles that can guide innovations resulting from advances in the biomedical and biotechnological fields as well as in clinical practice. Paediatric bioethics is a subfield that inquires into ethical dilemmas that arise in paediatric care, including issues related to informed consent or dissent regarding available treatments and end-of-life decisions.

The capacity of paediatric patients for self-determination regarding their healthcare and well-being is a topic of growing interest in the scientific literature. According to van Rooyen et al., determining the age and maturity at which a child can make autonomous decisions is complex [1]. A crucial aspect entails the role that should be given to their involvement in the decision-making process and the amount of information that should be shared with the paediatric patient regarding their diagnosis and prognosis. The complexity of this aspect is better understood when considering that paediatric patients do not usually have legal agency to make decisions for themselves. On the contrary, those holding parental responsibilities, such as parents or legal guardians, decide what course of treatment they would like to undergo and the level of the minor’s involvement in conversations [2]. Therefore, in the bioethical debate, the physicians’ role in considering the child’s wishes and preferences has been addressed. However, a consensus has not been reached among different Western countries. Healthcare providers may feel particularly distressed when the wishes of the child conflict with those of the parents or when the child disagrees with a clinically appropriate therapeutic option [3,4]

In many Western societies, parents assume the primary role of decision-makers for their child’s welfare. This assumption is supported by the common belief that parents will act in the child’s best interest. However, children should be encouraged to share their perspectives and should be involved in shared decision-making in a developmentally appropriate manner [5]. Indeed, even very young children may be able to understand the context of the illness and the value and weight of the medical treatments they undergo [6]. As cognitive abilities develop, this capacity gradually increases, reaching a stage where it is difficult not to assess it as complete, especially when the child is nearing the age of maturity and acquiring the associated rights [7]. Nonetheless, many adults feel unprepared to address topics such as illness, medical care, pain, and suffering with children. Hence, fostering education on the significance of open dialogue about these issues for children and adolescents is essential [8]. This is of pivotal importance not only in the presence of a sick child, but open dialogues on the topic of pain and suffering should be more broadly encouraged. Such an educational journey requires effective collaboration between parents and healthcare professionals, who can highlight the complexity of paediatric care thanks to their clinical expertise and experience [3].

Throughout the 20th century, thanks to advances in hygiene and medicine, the survival rate of paediatric patients with chronic or terminal illnesses improved significantly. However, this increase in life expectancy also increased morbidity rates among the paediatric population, leading to extended suffering [9]). Especially in younger children, the topic of suffering was often overlooked because it was commonly believed that children unable to communicate their suffering were not in pain at all [10].

Over time, a new cultural awareness has emerged, recognising that children, like adults, experience pain and suffering. This awareness has led to a growing focus on pain management and relief, contributing to the development of Paediatric Palliative Care (PPC). The availability of PPC has sparked debate on when to transition from curative treatments to a palliative approach [11]. PPC can be initiated to alleviate suffering, either alongside aggressive treatment or when such treatment becomes medically inappropriate and potentially harmful to the child. In certain situations, when the underlying condition is untreatable and the suffering becomes overwhelming, physicians, at the request of the parents and to avoid futile care, may consider withdrawing life-sustaining treatments. Although research in PPC has expanded significantly, there remains a shortage of evidence on key aspects such as decision-making, communication, and managing pain and symptoms, especially in children [12]. Consequently, the limited evidence makes formulating comprehensive recommendations challenging [13].

In recent decades, scientific progress in the paediatric field has increased the number of patients affected by complex pathologies and equally complex care and treatment pathways. Life expectancy has changed for many pathologies, but it has outlined life paths characterised by high care and treatment complexity. This has highlighted the need to involve parents and patients in the conscious and informed choice of care paths and shared planning.

### Aim of This Study

This study wants to explore the level of knowledge of a sample of modern Western society that relates to a minor subject in the growth path, who, for purely personal reasons or concerning a subject of the family or friends network, may be affected by an incurable disease with the need for a reflection of self-determination.

## 2. Materials and Methods

This study focused on Italian individuals of Caucasian ethnicity aged 18 years or older. The sample included a selection of demographic variables such as gender and age groups (<40 years, 40 ≤ 50 years, >50 years). Participants were also classified based on their educational qualifications, which included middle school diplomas, high school diplomas, university degrees, and postgraduate certificates. As for the participants’ professions, the categories included healthcare professionals, educators at school and university levels, legal experts, and others not included in these groups. Participants were also asked to indicate their parental status (yes or no).

A specific questionnaire was designed to collect this information, offering two response options (yes or no). This design was chosen because, to our knowledge, similar issues have not been examined in the literature. The questionnaire, which includes detailed questions about the concept of bioethics and personal and professional experiences relating to topics such as pain, incurability, and death in minors, among other specific issues, has been moved to Appendix A to allow greater focus on the description of methods in the main body of the manuscript. For a detailed view of the questions posed to participants, please refer to the manuscript’s Appendix A.

**Figure 1:**
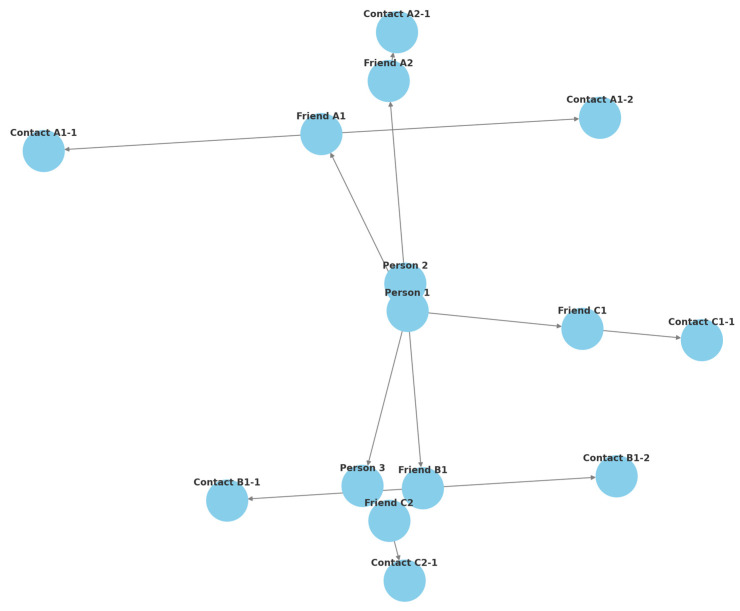
The diagram illustrates the dissemination of the online questionnaire through e-mail or WhatsApp. It begins with an initial group of known individuals and then expands hierarchically to include contact with friends.

The questionnaire was structured using Google Forms (https://docs.google.com/forms/u/0/ (accessed on 15 July 2021)) to be completed online. The survey was circulated through e-mail contacts (https://www.google.com/intl/en/gmail/about (accessed on 1 August 2021)) and WhatsApp (https://web.whatsapp.com/ (accessed on 1 August 2021)) for 1 month between August and September 2021. It started with an initial group of known individuals and then expanded hierarchically to include contacts of friends (Figure 1).

Subjects participated voluntarily after reading the information in Italian displayed on the first page of the online questionnaire. Since the subjects were not identifiable a priori, it was impossible to obtain formal individual consent.

Under these terms, our department and hospital did not need the study design approved by the local referral Ethics Committee.

### 2.1. Statistical Analysis

The information was downloaded from the online questionnaire structured using Google Forms in XLS file format and recorded in a Microsoft^®^ Excel^®^ database for Windows 11 (https://docs.google.com/forms/d/1vov5WcrAyBSraUPISNE8GBNnQwHTcO9rfRWuxVJzxQM/edit accessed on 30 September 2021). The data were statistically analysed using SPSS version 22.0 for Windows (SPSS Inc., Chicago, IL, USA).

Statistical analysis was performed using automatic clustering, which utilises the Bayesian Information Criterion (BIC) to determine the best number of clusters in the dataset. Pearson’s Chi-square test and Fisher’s exact test were employed to assess whether the distribution of variables among the clusters differed significantly. A *p*-value of <0.05 was deemed statistically significant.

### 2.2. Patient and Public Involvement

No patients or members of the public were involved in this study.

## 3. Results

Table 1 presents demographic data on survey respondents, including sex, parental status, age, educational qualifications, and job roles. Most participants are female (73.9%) and parents (65.8%). Age distribution is balanced, with 32.2% aged 18–40, 33.5% aged 40–50, and 34.3% over 50. Most respondents hold a degree (53.3%), followed by those with postgraduate qualifications (27.7%) and high school diplomas (17.4%). Regarding occupation, 44.7% work in healthcare, 10.2% are teachers, 4.4% are jurists, and 40.7% are employed in other fields.

Table 2 summarises responses to a survey on bioethical training and experiences related to minors, pain, incurability, and death. About half of the respondents (50.8%) reported receiving bioethics training, while nearly 48% had encountered bioethics issues involving minors. Over 61% had dealt with pain, incurability, or death professionally or personally, and 58.8% specifically concerning minors. Most (98.5%) believed adults should receive bioethical training on these issues, but only 25.3% had undergone such training. Most respondents (88.5%) favoured a shared programme involving parents, schools, and paediatricians for bioethical education. Similarly, 85.3% supported shared responsibility for fostering self-determination in therapeutic choices. Regarding timing, 73.8% suggested addressing these issues gradually from childhood to adolescence. Only 1.6% felt they should not be discussed with minors.

The results of Table 3 present a cluster analysis based on the Bayesian Schwarz Criterion (BIC).

The analysis was conducted with progressively increasing clusters, ranging from 1 to 5. The BIC values consistently decrease as the number of clusters increases, reflecting an improvement in model quality. The most substantial reduction in BIC occurs when moving from one to two clusters (−1606.117), with progressively smaller decreases for additional clusters.

The BICa Change metric, a normalised measure of relative improvement, indicates that the benefit of adding clusters diminishes as the number of clusters grows, starting with a maximum value of 1.000 for the transition from one to two clusters. Similarly, the distance between clusters, reported in the distance measurement report, shows a decreasing trend as the number of clusters increases, starting from 2.477 (one to two clusters) and dropping to 1.238 (four to five clusters).

Finally, the results suggest that moving from one to two clusters is the most significant improvement. Beyond this point, adding more clusters yields diminishing returns, with an optimal solution likely lying between two and three clusters.

Table 4 presents the distribution of categorical variables between the two clusters. This study reveals significant statistical differences between the two clusters, indicating a clear distinction in sociodemographic, educational, and experiential characteristics.

Cluster 1 exhibits a higher prevalence of females (78.3% vs. 68.5%), whereas Cluster 2 has a higher prevalence of males (31.5% vs. 21.7%; *p* = 0.001). Most parents are found in Cluster 2 (75.1%), compared to 58.3% in Cluster 1 (*p* < 0.001). Cluster 1 also shows a more excellent representation of individuals with a higher education level (postgraduate 38.6% vs. 14.1%), while Cluster 2 has a predominance of lower educational skills (middle school 3.0% vs. 0.4%; *p* < 0.001).

Individuals under 40 are more prominently represented in Cluster 1 (37.4% vs. 25.7%), while Cluster 2 displays a more uniform distribution across age groups (*p* = 0.001). Furthermore, Cluster 1 includes most individuals with training in bioethics (81.5% vs. 12.8%; *p* < 0.001).

The majority in Cluster 1 also possess experience with bioethical issues concerning minors (81.1%), while those lacking such experience predominantly reside in Cluster 2 (93.5%; *p* < 0.001). Additionally, Cluster 1 comprises more individuals with personal or professional experience with minors (81.1%), contrasting with Cluster 2, where 93.5% lack this experience (*p* < 0.001).

An overwhelming 99.8% of individuals in Cluster 1 find adult bioethics helpful training, compared to 97.0% in Cluster 2 (*p* < 0.001). Almost all participants who received personal training on these topics belong to Cluster 1 (45.1%), while those without training are mainly found in Cluster 2 (99.2%; *p* < 0.001).

Cluster 1 prefers training to be shared among various adults (92.3% vs. 83.9%; *p* = 0.001) and supports a collaborative approach to developing self-determination (90%), with a significant difference compared to Cluster 2 (79.3%; *p* < 0.001). Lastly, Cluster 1 is more inclined to address these issues gradually from childhood (84.3% vs. 60.7%; *p* < 0.001).

## 4. Discussion

The analysis of the two adult groups highlights how differences in sociodemographic, educational, and experiential profiles significantly impact bioethical approaches to sensitive issues such as pain and death in minors.

Cluster 1 predominantly comprises women (78.3% vs. 68.5%). This group is characterised by a higher level of education (38.6% with postgraduate degrees compared to 14.1%). They demonstrate greater sensitivity towards bioethical issues. This group has a solid foundation in bioethics (81.5% vs. 12.8%) and direct experiences with pain, incurability, and death (75.2% vs. 38.5%). These factors lead them to engage with these topics proactively and informally. Their sensitivity is reflected in a preventive approach to bioethical education. They emphasise the necessity of engaging in conversations concerning these topics during childhood. Most of this group believes that gradual education on topics related to pain and suffering is essential for developing self-determination skills in minors. They advocate for collaboration among families, schools, and paediatricians (90.0% vs. 79.3%) to increase minors’ awareness and empower them to exercise self-determination. This approach indicates a clear preference for early and structured educational intervention. According to this group, implementing structured educational programmes not only protects children from potential future trauma but also prepares them to navigate complex situations autonomously and competently.

On the other hand, Cluster 2 is predominantly composed of men (31.5% vs. 21.7%) and individuals with lower educational backgrounds. This group exhibits a more reactive attitude towards bioethical quandaries. Interviewed members of this group report having engaged in fewer opportunities related to specific training in bioethics and fewer direct experiences with topics such as pain and death. Consequently, they only address these topics when specific situations arise. This reactive approach focuses less on prevention and more on resolving immediate crises. There is a greater tendency within this group to delegate the management of these issues to parents or paediatricians rather than encouraging a shared responsibility among all parties involved.

Overall, our study identified a clear distinction between two groups of adults, showing how their different perspectives influence their positions regarding involving minors in conversations about death and suffering. Their sociodemographic, educational, and experiential profiles differ significantly. This has important implications when it comes to their approaches towards bioethical considerations. These two contrasting perspectives underscore the need for targeted educational interventions.

To our knowledge, limited research focuses on targeted educational interventions and strategies that can encourage the development of educational programmes in bioethics. This lack of research also entails scant empirical studies that can grasp adult perceptions on educating children on bioethical topics [14].

Despite this lack of research, pain and suffering are central issues in paediatric bioethics. Taking care of a sick child involves delicate choices that profoundly impact the well-being of minors and their families. In this context, paediatric bioethics can provide ethical principles and practical recommendations that enhance parental decision-making [4] and foster children’s involvement in decision-making.

Disagreements between physicians and families about end-of-life decisions generate emotionally challenging situations [15]. Despite the significant variability among different countries, institutions, and family preferences, the paediatric population is often shielded from engaging in conversations that may create discomfort, such as those regarding the topic of pain and suffering. Although it would be ideal that children would never have to discuss topics related to pain and suffering, many times, they are either directly or indirectly exposed to them. Therefore, there is a need to implement suitable support and training for the paediatric population in the context of ethical shared decision-making [16].

Understanding how sociodemographic and educational backgrounds influence perceptions and approaches to such topics [17,18] helps develop targeted educational programmes [19] with the hope of increasing the overall knowledge of bioethical issues among the general population, including minors [17,18,19]. These opportunities are necessary for growing skills in appreciating ethical challenges among children [3].

Having early conversations with parents on the topics of pain and suffering provides them with the tools to navigate complex ethical and medical situations. Although it is assumed that parents should be given precedence in disagreements regarding treatment choices for a sick child, healthcare professionals play a significant role in educating the parents and empowering them to make informed decisions [20]. Hence, incorporating educational programmes that effectively address bioethical issues is crucial for assisting parents in their role as ethical decision-makers. Nevertheless, many healthcare professionals feel inadequately prepared to handle complex ethical situations.

In our study, the first group of adults highlights the importance of ongoing education in this field. A high percentage of individuals with specific training in bioethics and significant experiences with complex issues such as pain, death, and incurability characterise this group. As a result, they adopt a more conscious and proactive approach to ethical questions. They prefer discussing topics related to pain and suffering through formal and informal conversations with children during childhood. Their standpoint reflects a preventive approach that aims to provide instruments for self-determination through coordinated involvement among family, school, and paediatricians. In contrast, the second adult group has engaged in less specific training in bioethics, which is associated with a lower education background and older age. This difference can profoundly influence their ability to address ethical issues, limiting their understanding and active participation in their children’s healthcare decision-making processes.

Aligned with previous studies that have emphasised how gender, age, educational level, and cultural context significantly impact ethical perspectives [21,22], this study shows that gender differences may need to be considered when delineating ways to engage in bioethical conversations with minors. The examined sample shows that the first group of adults (Cluster 1), predominantly composed of women (58.6%), exhibits greater sensitivity to these issues compared to the second group (Cluster 2), which has a higher male representation. This disparity suggests that gender may play a role in defining ethical standpoints.

Our study found that the first group of adults is younger, with 37.4% of individuals under 40 years old. In contrast, the second group has fewer individuals younger than 40 (25.7%). The literature suggests that younger individuals tend to have different opinions on ethical dilemmas than older adults. Young adults often adopt a more flexible and open approach to innovation, emphasising individual rights and personal autonomy. Conversely, older adults may tend to prioritise community and collective values. The survey revealed a clear distinction between the two adult groups based on their education level. The first group predominantly comprises individuals with postgraduate education (38.6% vs. 14.1%) and fewer with middle school certificates (0.4% vs. 2%).

Furthermore, the second adult group is characterised by a higher percentage of parents (75.1%) than the first group (58.3%). Parents play a central role in the ethical education of their children [23]. They are often called upon to make fundamental decisions regarding their children’s health and well-being, given that they cannot fully exercise their autonomy [7]. The results of this survey are, therefore, worth noting. Our results suggest that parents may be less inclined to have conversations with children regarding pain and suffering. Further studies are needed to elucidate the reasons behind this position and what strategies may be implemented to increase awareness among parents.

This study is not free from limitations. Using binary responses (yes/no) can lead to an excessive simplification of the information collected. Moreover, distributing the questionnaire through personal networks may not ensure adequate representation of the entire population. Additionally, a 1-month data collection period might not obtain significant opinion variations. Furthermore, personal interests in bioethical topics could influence the motivation to participate, leading to a sample with more pronounced opinions. Finally, circumstances related to the COVID-19 pandemic may have impacted respondents’ perceptions, but this study did not consider such factors.

Despite its limitations, this study presents several strengths. The scarcity of specific questions in the previous literature gives this research an innovative character in the paediatric context. Including adult Caucasian Italian residents allows for the analysis of a homogeneous sample. Additionally, considering demographic variables such as gender, age, and profession contributes to a better understanding of the variety of approaches towards initiating conversations with minors on topics such as pain and suffering. The arrangement of the questionnaire, characterised by binary responses and multiple-choice options, accelerates precise data collection and favours practical statistical analysis. The implementation of online platforms for data collection develops participation possibilities, contributing to the amplified validity of the results. However, the study conducted using accurate statistical techniques guarantees the reliability of the results discussed.

Finally, in interpreting these results, it is crucial to note that the distinctions between the two clusters do not indicate biases or stereotypes but highlight the diversity of experiences in bioethics. Recognising these differences allows for developing more targeted educational strategies sensitive to individual contexts and needs. By focusing on tailored interventions, we aim to promote a practical approach to bioethical education, ensuring that it is adapted to the varied realities of individuals and enhances their engagement

## 5. Conclusions

In conclusion, our study has uncovered the existence of two distinct groups of adults, each with a different approach to bioethical issues in paediatrics, differentiated by specific sociodemographic, educational, and experiential profiles. This distinction is significant for clinical practice, as education and personal experiences significantly influence the inclination to tackle ethical issues with minors. The first group, equipped with a higher education level and extensive bioethics experience, adopts a proactive approach. These adults promote anticipatory and collaborative bioethical education, actively involving families, schools, and health professionals. In clinical practice, this approach can translate into preventive and structured educational interventions aimed at sensitising minors to complex topics such as pain, incurability, and death. The second group, with less advanced training and less direct exposure to bioethical issues, tends to adopt a reactive approach, addressing such themes only in critical situations. This behaviour underscores the need for healthcare professionals to adopt direct and specific communication strategies, providing active support in the face of ethical dilemmas. Understanding these differences in adult profiles can guide the development of tailored educational programmes to enhance bioethical competencies in paediatric clinical practice. Implementing these interventions could improve communication between professionals, families, and minors, facilitate informed decisions, and promote adherence to agreed-upon care programmes.

## Figures and Tables

**Table 1:** Demographic traits of the sample involved in the analysis (N = 889). The number of participants (n) and the percentage (%) are reported for each category.

Demographic Variable	Categories	Frequency, n. (%)
N.	Participants	889
Sex	Male	232 (26.1)
Parent	Yes	585 (65.8)
		
Age (years range)	>18 ≤ 40	286 (32.2)
	>40 ≤ 50	298 (33.5)
	>50	305 (34.3)
		
Educational qualification	Middle school certificates	14 (1.6)
	High school diplomas	155 (17.4)
	University degrees	474 (53.3)
	Postgraduate certificates	246 (27.7)
		
Job	Healthcare professionals	397 (44.7)
	Educators	91 (10.2)
	Jurists	39 (4.4)
	Other	362 (40.7)

**Table 2:** Responses of the interviewed adults (N = 889) to questions regarding training and personal and professional experience on paediatric bioethics topics. The number of participants (n) and the corresponding percentage of those who answered affirmatively are reported for each question.

Questions Answered in the Affirmative	N. (%)
Have you ever received training on the concept of bioethics?	452 (50.8)
In your personal and/or professional experience, have you ever had to deal with bioethics issues concerning a minor?	425 (47.8)
In your personal and/or professional experience, have you ever had to deal with issues concerning pain, incurability and/or death?	545 (61.3)
In your personal and/or professional experience, have you ever had to deal with issues concerning pain, incurability, or death with a minor?	523 (58.8)
Do you think it would be helpful for an adult to have bioethical training on issues concerning pain, incurability, and/or death?	876 (98.5)
Have you ever personally received training on these issues?	225 (25.3)
**Who do you think should provide training on the issues of pain, incurability, and death?**	
Parents or guardians in the home environment	45 (5.1)
Educational institution	30 (3.4)
Family paediatrician in prevention visits (health assessments)	27 (3)
All of the above options with a shared programme	787 (88.5)
**Who should provide the tools for developing self-determination and awareness of shared therapeutic choice?**	
Parents or guardians in the home environment	52 (5.8)
Educational institution	24 (2.7)
Family paediatrician in prevention visits (health assessments)	55 (6.2)
All of the above options with a shared programme	758 (85.3)
**When do you think it is time to address the issues of pain, incurability, and death with a minor?**	
In the event of a personal or personal event	128 (14.4)
Gradually, from childhood to adolescence	656 (73.8)
In the adolescent period	91 (10.2)
These are issues that should not be addressed with a minor	14 (1.6)

**Table 3:** The statistical analysis presents the results of automatic clustering using the Bayesian Information Criterion (BIC) to determine the optimal number of clusters within a dataset. The variables included are those represented in Table 1 and Table 2. The change ratios pertain to the solution with two clusters.

Number of Clusters	Bayesian Schwarz Criterion (BIC)	BICa Modification	BICb Change Report	Distance Measurement Report
1	15,441.010			
2	13,834.893	−1606.117	1.000	2.477
3	13,275.489	−559.404	0.348	1.446
4	12,934.788	−340.700	0.212	1.163
5	12,662.679	−272.109	0.169	1.238

**Table 4:** The table presents the distribution of categorical variables between the two clusters. The associated statistical results (Pearson’s Chi-square or Fisher’s exact tests) assess the significance of the observed differences between the clusters.

Variable	Cluster 1 (%)	Cluster 2 (%)	Chi-Squared Person	Fisher’s Exact Test
n.	492	397		
Gender (M = 1, F = 2)				
M	21.7	31.5		
F	78.3	68.5	10.803	0.001
Are you a parent? (0 = No, 1 = Yes)				
No	41.7	24.9		
Yes	58.3	75.1	27.328	<0.001
Educational qualification				
Middle school certificates	0.4	3.0		
High school	10.2	26.4		
University	50.8	56.4		
Postgraduate	38.6	14.1	91.975	<0.001
Age categories (<40, 40–50, >50 years)				
<40	37.4	25.7		
40–50	29.9	38		
>50	32.7	36.3	14.526	0.001
1. Have you ever received training in bioethics?				
No	18.5	87.2		
Yes	81.5	12.8	414.397	<0.001
2. In your personal and/or professional experience, have you ever had to deal with bioethics issues concerning a minor?				
No	18.9	93.5		
Yes	81.1	6.5	489.359	<0.001
3. In your personal and/or professional experience, have you ever had to deal with issues concerning pain, incurability and/or death?				
No	11.6	72.3		
Yes	88.4	27.7	341.332	<0.001
4. In your personal and/or professional experience, have you never dealt with issues concerning pain or incurability with a minor?				
No	24.8	61.5		
Yes	75.2	38.5	121.944	<0.001
Do you think it would be helpful for an adult to have bioethical training on issues concerning pain, incurability and death?				
No	0.2	3.0		
Yes	99.8	97.0	12.121	<0.001
Have you ever personally received training on these issues?				
No	54.9	99.2		
Yes	45.1	0.8	228.777	<0.001
Who do you think should provide training on the issues of pain, incurability and death?				
Parents or guardians in the home environment	2.6	8.1		
Educational institution	2.6	4.3		
Family paediatrician in prevention visits (health assessments)	2.4	3.8		
All the above options with a shared programme	92.3	83.9	14.541	0.001
Who should provide the tools for developing self-determination and awareness of shared therapeutic choice?				
Parents or guardians in the home environment	4.3	7.8		
Educational institution	1.8	3.8		
Family paediatrician in prevention visits (health assessments)	3.9	9.1		
All of the above options with a shared programme	90.0	79.3	20.373	<0.001
When do you think it is time to address the issues of pain, incurability and death with a minor?				
In the event of a personal or personal event	10.2	19.6		
Gradually, from childhood to adolescence	84.3	60.7		
In the adolescent period	4.7	17.1		
These are issues that should not be addressed with a minor	0.8	2.5	67.723	<0.001

## Data Availability

The datasets presented in this article are not readily available because the data are part of an ongoing study.

## Appendix A

The addendum section reports the questionnaire in English and Italian language.

**Variables**	**Variabili**
n.	n.
Geder (Male = 1, Female = 2)	Genere (Maschio = 1, Femmina = 2)
M	M
F	F
Are you a parent? (0 = No, 1 = Yes)	E’ genitore? (0 = No, 1 = Si)
No	No
Yes	Si
Educational qualification	Titolo di studio
Middle school certificates	Diploma di scuola media
High school diplomas	Scuola superiore/Liceo
University degrees	Laurea
Postgraduate certificates	Postlaurea
Age (<40, 40–50, >50 years)	Età (<40, 40–50, >50 anni)
<40	<40
40–50	40–50
>50	>50
Have you ever received training on the concept of bioethics?	1. Hai mai ricevuto una formazione in bioetica?
No	No
Yes	Sì
In your personal and professional experience, have you ever had to deal with bioethical issues concerning a minor?	2. Nella sua esperienza personale e/o professionale ha mai dovuto affrontare temi di bioetica riguardanti un minore?
No	No
Yes	Sì
In your personal and/or professional experience, have you ever had to deal with issues related to pain, incurability, and/or death concerning a minor?	3. Nella tua esperienza personale e/o professionale, hai mai avuto a che fare con tematiche riguardanti il dolore, l’inguaribilità e/o la morte?
No	No
Yes	Sì
In your personal and/or professional experience, have you ever had to address issues related to pain, incurability, and death with a minor?	4. Nella tua esperienza personale e/o professionale, non ti sei mai occupato di questioni riguardanti il dolore o l’incurabilità con un minore?
No	No
Yes	Sì
Do you consider it useful for an adult to receive bioethical training on topics related to pain, incurability, and/or death?	Ritiene utile per un soggetto adulto avere una formazione bioetica sui temi riguardanti dolore, inguaribilità e/o morte?
No	No
Yes	Sì
Have you personally received training on these topics?	Ha mai ricevuto personalmente una formazione su questi temi?
No	No
Yes	Sì
Who do you think should provide training on pain, incurability, and death?	Chi pensi dovrebbe fornire formazione sui temi del dolore, dell’incurabilità e della morte?
Parents or guardians in the home environment	Genitori o tutori nell’ambiente domestico
Educational institutions	Istituto di istruzione
Family paediatricians during preventive visits (health check-ups)	Pediatra di famiglia nelle visite di prevenzione (accertamenti sanitari)
All of the above options with a shared programme	Tutte le opzioni di cui sopra con un programma condiviso
Who should provide the tools for developing self-determination and awareness of common therapeutic choices?	Chi dovrebbe fornire gli strumenti per sviluppare l’autodeterminazione e la consapevolezza di una scelta terapeutica condivisa?
Parents or guardians in the home environment	Genitori o tutori nell’ambiente domestico
Educational institutions	Istituto di istruzione
Family paediatricians during preventive visits (health check-ups)	Pediatra di famiglia nelle visite di prevenzione (accertamenti sanitari)
All of the above options with a shared programme	Tutte le opzioni di cui sopra con un programma condiviso
When do you believe is the right time to discuss topics of pain, incurability, and death with a minor?	Quando pensi che sia il momento di affrontare i temi del dolore, dell’incurabilità e della morte con un minore?
In the case of a personal event or related to their sphere	In caso di evento personale o personale
Gradually from childhood to adolescence	Gradualmente, dall’infanzia all’adolescenza
During adolescence	Nel periodo adolescenziale
These topics should not be discussed with a minor	Questi sono problemi che non dovrebbero essere affrontati con un minore
